# Effect of a Combination of *Lactiplantibacillus plantarum* KC3 and *Leonurus japonicus* Extracts in Respiratory Discomfort: A Randomized, Double-Blind, Placebo-Controlled Trial

**DOI:** 10.3390/nu16132128

**Published:** 2024-07-03

**Authors:** Hyung-jin Kim, Jun-Ho Lee, So-Young Kwon, Yoo Jin Kwon, Mi-Sun Park, Kyung Hwan Kim, Chang Hun Shin, Jong-Cheon Joo, Soo-Jung Park

**Affiliations:** 1 College of Korean Medicine, Woosuk University, Jeonju-si 54986, Jeollabuk-do, Republic of Korea; dorunnom@gmail.com (H.-j.K.); celtece@hanmail.net (J.-H.L.); 2 CKD BiO Research Institute, 8 Chungjeong-ro, Seodaemun-gu, Seoul 03742, Republic of Korea; ksy2022@ckdbio.com (S.-Y.K.); kkyyjj8@ckdbio.com (Y.J.K.); pms@ckdbio.com (M.-S.P.); kimkh@ckdbio.com (K.H.K.); chshin@ckdbio.com (C.H.S.); 3 Department of Constitutional Medicine, College of Korean Medicine, Wonkwang University, Iksan-si 54538, Jeollabuk-do, Republic of Korea; 4 Department of Sasang Constitutional Medicine, College of Korean Medicine, Woosuk University, Jeonju-si 55338, Jeollabuk-do, Republic of Korea

**Keywords:** *Lactiplantibacillus plantarum* KC3, chronic obstructive pulmonary disease, probiotic bacteria, gut–lung axis

## Abstract

The increased global prevalence of chronic respiratory diseases in recent years has caused a substantial public health burden. *Lactiplantibacillus plantarum* KC3 and *Leonurus japonicus* Houtt. (LJH) extracts can alleviate respiratory symptoms and improve lung function in vitro and in vivo. However, the clinical efficacy and safety profile of this combination in patients with respiratory diseases remain unclear. Therefore, this multicenter, randomized, double-blind, placebo-controlled clinical trial aimed to evaluate the efficacy and safety of *L. plantarum* KC3 and LJH extracts in adults with respiratory discomfort. This mixture was termed ‘CKDB-315’. Participants, randomly assigned to the CKDB-315 or placebo groups, were treated for 12 weeks. Assessments included the St. George’s Respiratory Questionnaire (SGRQ) and the Chronic Obstructive Pulmonary Disease Assessment Test (CAT). The CKDB-315 group showed considerably improved SGRQ and CAT scores compared with the placebo group. Secondary outcomes, including dyspnea, pulmonary function, total antioxidant status, and inflammatory cytokine levels, were consistent with the primary outcomes. Exploratory analyses of the gut microbiota and short-chain fatty acid contents revealed the potential mechanisms underlying the effects of CKDB-315. Finally, safety analysis indicated that CKDB-315 was well tolerated and caused few adverse events. Our findings indicate that CKDB-315 is a promising therapeutic option for respiratory discomfort in adults.

## 1. Introduction

Chronic respiratory diseases (CRDs) such as chronic obstructive pulmonary disease (COPD), asthma, bronchitis, and respiratory infections are a serious public health challenge worldwide [1]. According to the 2019 Global Burden of Disease, Injuries, and Risk Factor Study, which provides the most comprehensive measure of the epidemiological characteristics of noncommunicable diseases, CRDs have a global prevalence of approximately 454.6 million cases and cause 4 million deaths annually. These diseases are the third leading cause of death worldwide [2] and place a considerable burden on individuals, families, and healthcare systems, due to their high prevalence, associated morbidity, and mortality [3]. The incidence of respiratory diseases has recently increased because of the coronavirus disease (COVID-19), worsening air pollution, and an aging population [4]. In particular, symptoms of underlying respiratory diseases worsen under high fine dust concentrations [5,6]. Despite advances in medical research and the development of treatment modalities, the management of respiratory diseases remains complex and their multifactorial nature often requires innovative treatment approaches.

The potential therapeutic role of probiotics and plant-derived extracts in improving respiratory health has garnered substantial interest [7,8]. Probiotics, live microorganisms primarily comprising bacteria and yeast, confer health benefits when consumed adequately. Probiotics are commonly found in fermented foods, such as yogurt, kefir, and kimchi, as well as in dietary supplements. They promote gut health by restoring and maintaining a healthy balance of intestinal microflora [9]. Moreover, probiotics modulate the gut–lung axis by influencing immune function and inflammatory responses in the gut and lungs [10,11]. These beneficial bacteria can migrate from the gut to the respiratory tract, interact with immune cells, and regulate the immune defense against respiratory pathogens and allergens [12,13]. Disruptions in the gut microbiota composition, such as dysbiosis, can negatively impact respiratory health and exacerbate lung conditions such as asthma, chronic obstructive pulmonary disease (COPD), and respiratory infections [14]. *Lactiplantibacillus plantarum* KC3 (formerly *Lactobacillus plantarum*) (*L. plantarum* KC3) is a probiotic strain that modulates immune responses and reduces airway inflammation, potentially contributing to the alleviation of respiratory disease symptoms and improved patient outcomes [15].

*Leonurus japonicus* Houtt. (LJH), commonly known as motherwort, is a traditional Chinese medicine used to treat edema, ulcers, and gynecological disorders [16,17]. LJH contains active components with diverse biological effects, including anticancer, analgesic, antioxidant, anti-inflammatory, and neuroprotective properties [16]. The potential synergistic effects of *L. plantarum* KC3 combined with LJH extract in alleviating respiratory symptoms and improving lung function have been reported [18]. However, despite promising preclinical evidence, the clinical efficacy and safety profile of this combination in patients with respiratory diseases are yet to be comprehensively evaluated.

CKDB-315 is a novel intervention that combines *L. plantarum* KC3 with LJH extract, offering a synergistic approach for respiratory disease management by harnessing the potential benefits of probiotics and natural compounds. Therefore, this study aimed to assess the efficacy and safety of CKDB-315 in patients with respiratory discomfort through to a rigorous multicenter, randomized, double-blind, placebo-controlled clinical trial. The present study provides valuable insights into the therapeutic effects of CKDB-315 and its potential role in reshaping the treatment landscape of respiratory diseases. These findings may inform clinical practice and guide the development of novel therapeutic strategies aimed at improving the lives of individuals with respiratory diseases.

## 2. Materials and Methods

### 2.1. Study Design

This research was a multicenter, randomized, double-blind, placebo-controlled clinical trial carried out between September 2022 and April 2023 at two locations: Wonkwang University Oriental Medical Hospital and Woosuk University Korean Medicine Medical Center. The study protocol and its revisions received approval from the institutional review boards at both sites (IRB approval No.: WUJKMH-IRB-2022-0006 and WSOH IRB H2206-01-01). The trial adhered to the Helsinki Declaration and Good Clinical Practice (GCP) guidelines, and was registered with the Clinical Research Information Service (CRIS, http://cris.nih.go.kr, registered on 12 April 2023, clinical trial No.: KCT0008407).

### 2.2. Participants

Eligible participants were required to be aged between 19 and 75, have experienced persistent respiratory discomfort symptoms (such as cough, phlegm, or shortness of breath) for at least 12 weeks prior to screening, and be nonsmokers (including those who had quit smoking at least 6 months before screening). The exclusion criteria consisted of: (1) women who were pregnant or breastfeeding; (2) individuals with radiological evidence suggesting the presence of other respiratory conditions that could be contributing to their current symptoms; (3) individuals with a post-bronchodilator forced expiratory volume in one second (FEV1) to a forced vital capacity (FVC) ratio of less than 0.7, or an increase in FEV1 of 12% or more and greater than 200 mL after using a bronchodilator; (4) notable respiratory conditions such as chronic obstructive pulmonary disease, asthma, ongoing lung infection, severe bronchiectasis, pulmonary fibrosis, or interstitial lung disease; (5) ongoing significant infections within the 12 weeks prior to screening; (6) autoimmune diseases that necessitate immunosuppressive treatment; (7) a history of substance or alcohol abuse; (8) notable conditions affecting the cardiovascular, gastrointestinal, liver, kidney, nervous system, musculoskeletal system, infectious diseases, endocrine system, metabolic system, blood, immune system, mental health, or major physical injuries; (9) prior use of medications and health-functional foods that affect respiratory health within the 3 months before screening; (10) regular consumption of probiotics, dietary fiber, fructooligosaccharides, synbiotics, or fermented dairy products within the 4 weeks before screening; (11) allergic reaction to the investigational product or its components; (12) serum creatinine levels equal to or greater than twice the upper limit of normal (ULN) or aspartate transaminase (AST)/alanine transaminase (ALT) levels equal to or greater than three times ULN; (13) participation in other clinical trials within the 30 days before screening; (14) other reasons determined by the investigator as unsuitable for participation. Women who could potentially become pregnant were obligated to have a negative pregnancy test and consent to using a dependable form of birth control. Prior to their participation in the trial, all individuals supplied written consent after being fully informed.

### 2.3. Randomization

The eligible participants were randomly assigned to either the CKDB-315 group or the placebo group in a 1:1 ratio. This assignment was based on a code created by the block randomization approach using SAS (version 9.4, SAS Institute, Cary, NC, USA). Stratification of the site was implemented during the randomization process. The investigators and volunteers were kept unaware of the investigational product’s identity throughout the trial. 

### 2.4. Intervention

The CKDB-315 supplement was formulated by Chong Kun Dang BiO Corp., a company based in Ansan, Republic of Korea. The CKDB-315 group was administered CKDB-315 capsules, each containing 60 mg of *Lactiplantibacillus plantarum* KC3 and 266.67 mg of *Leonurus japonicus* Houtt. extracts. The ethanol extract of *Leonurus japonicus* Houtt. was obtained from KT&G R&D Headquarters (Daejeon, Repulic of Korea). Briefly, *Leonurus japonicus* Houtt. was harvested in Yeongcheon, Gyeongsangbuk-do, and the above ground part was extracted with 30% (*v*/*v*) ethanol, filtered, condensed, and spray-dried. The placebo capsule was indistinguishable in appearance from the active one. Participants were directed to consume one capsule thrice day for a duration of 12 weeks. The study visit took place at weeks 6 and 12 following randomization.

### 2.5. Efficacy Outcomes

In order to assess Health-Related Quality of Life (HRQL), we employed the St. George’s Respiratory Questionnaire (SGRQ) and the COPD Assessment Test (CAT), both of which are specialized tools for measuring respiratory health. The SGRQ consists of 50 questions divided into three domains: symptoms, activities (limitations), and impacts (of disease). These questions are used to create a score ranging from 0 (indicating the best condition) to 100 (indicating the worst condition). The least clinically significant difference (MCID) for the total score of the St. George’s Respiratory Questionnaire (SGRQ), which is widely acknowledged as a sign of improvement from the initial state, is a reduction of 4 units. The CAT comprises a total of eight components. The score for each item falls within the range of 0–5 points. This contributes to a total CAT score that can range from 0 to 40 points. Higher scores indicate a poorer health state. A change in score of −2 points is considered the minimum clinically important difference (MCID). 

The assessment of dyspnea was conducted using the modified Medical Research Council (mMRC) and the dyspnea visual analogue scale (DVAS). The mMRC dyspnea scale is graded on a range of 0–4, based on the intensity of the dyspnea. The categorical assessment of change from baseline in mMRC was determined by classifying patients who experienced a decrease of 1 grade as ‘Improved’ and those who saw an increase of 1 grade as ‘worsened’. The DVAS is a vertical line measuring 100 mm. It is labeled at 0 mm as ‘Not breathless at all’ and at 100 mm as ‘Worst possible breathlessness’. Fasting blood samples were collected in order to evaluate the total antioxidant status (TAS) and levels of inflammatory cytokines (hs-CRP, TNF-α, IL-6). Total antioxidant status (TAS) was determined using colorimetry with commercially available kits (TAS Assay Kit, Mega tip, Gaziantep, Turkey). Levels of hs-CRP, TNF-α, and IL-6 were measured using enzyme-linked immunosorbent assay (ELISA) kits (CRPHS for hs-CRP, Roche, Mannheim, Germany; Human TNF-α HS ELISA for TNF-α, R&D, Minneapolis, MN, USA; Human IL-6 HS ELISA for IL-6, R&D, Minneapolis, MN, USA). The main outcomes measured were the differences in SGRQ total score and CAT score between the beginning and the conclusion of the 12-week period. The secondary endpoints assessed in this study included the following measurements: the change in SGRQ domain scores (Symptoms, Activity, Impacts); the proportion of subjects who achieved a 4-point reduction in SGRQ total score; the proportion of subjects who achieved a 2-point reduction in CAT score; the categorized change in mMRC dyspnea scale; the change in DVAS; the changes in pulmonary function values (FVC, predicted FVC%, FEV1, predicted FEV1%, FEV1/FVC); the change in TAS; and the changes in inflammatory cytokine levels (hs-CRP, TNF-α, IL-6). The efficacy outcomes were assessed at the beginning of this study and 12 weeks after the treatment.

### 2.6. Safety Outcomes

The safety outcomes encompassed voluntarily reported adverse events (AEs), results from clinical laboratory tests, vital signs, and observations from chest X-rays and physical examinations. The coding of all adverse events (AEs) was performed in accordance with the Medical Dictionary for Regulatory Activities (MedDRA Version 26.0). The AEs were then categorized based on the system organ class (SOC) and preferred term (PT).

### 2.7. Exploratory Outcomes: Fecal Microbiome Analysis 

An investigation was conducted to investigate the changes in the gut microbiota and metabolome from baseline to week 12. The microbiome assessment involved isolating total genomic DNA from fecal samples using the DNeasy 96 PowerSoil Pro QiaCube HT kit (QIAGEN, Hilden, Germany). Barcode fusion primers were used to amplify the V3–V4 region of the bacterial 16S rRNA gene. The amplicon obtained was purified using HiAccuBead (AccuGene, Incheon, Republic of Korea) and subjected to analysis on the 4200 TapeStation System (Agilent, Palo Alto, CA, USA). The sequencing was performed using the Ion GeneStudio™ S5 System (Thermo Fisher Scientific, Waltham, MA, USA) following the manufacturer’s instructions. The sequencing data were transformed into FASTQ files for analysis, using the QIIME2 pipeline (https://qiime2.org/, accessed on 1 August 2023) for sequence analysis and basic statistics. The FASTQ data were subjected to quality filtering and trimming using the QIIME 2 DADA2 plugin. Taxonomic classification was carried out using the Greengenes2 database (version 2022.10). The assessment of gut bacteria diversity was conducted using Shannon’s index and Pielou’s evenness index. A pairwise difference test was used to compare the changes in alpha diversity over time. The analysis of short-chain fatty acids (SCFAs) in fecal samples was conducted at the Korea Research Institute of Biomedical Science (KRIBS) using the 7890 A gas chromatography–mass spectrometry system (Agilent, Palo Alto, CA, USA) with flame ionization detection. The system was equipped with a DB-FATWAX Ultra Inert column (30 m, 0.32 mm, 0.25 μm, Agilent, Palo Alto, CA, USA). The statistical analyses were conducted using GraphPad Prism 10.0.0.

### 2.8. Statistical Analysis

The results are reported as the mean ± standard deviation (SD), mean ± standard error (SE), or as counts with percentages, depending on the type of data. To compare continuous variables between groups, either an independent *t*-test or Wilcoxon’s rank-sum test was used. The categorical variables were analyzed using either Fisher’s exact test or Pearson’s chi-squared test. Paired *t*-tests or Wilcoxon’s signed rank tests were used to compare within groups. Furthermore, this study employed analysis of covariance (ANCOVA) with adjustments for baseline values to assess the differences between the groups in terms of changes from the first measurements. The statistical analyses were performed using SAS software (version 9.4, SAS Institute, Cary, NC, USA). A two-sided *p*-value below 0.05 was deemed statistically significant. The efficacy analyses were conducted using the Full Analysis Set (FAS) based on the ‘Intention-to-treat Principle’ (ITT). The safety analyses, on the other hand, were performed using the Safety Analysis Set (SS) based on the actual treatment received.

## 3. Results

### 3.1. Sub Characteristics of the Participants

**Figure 1:**
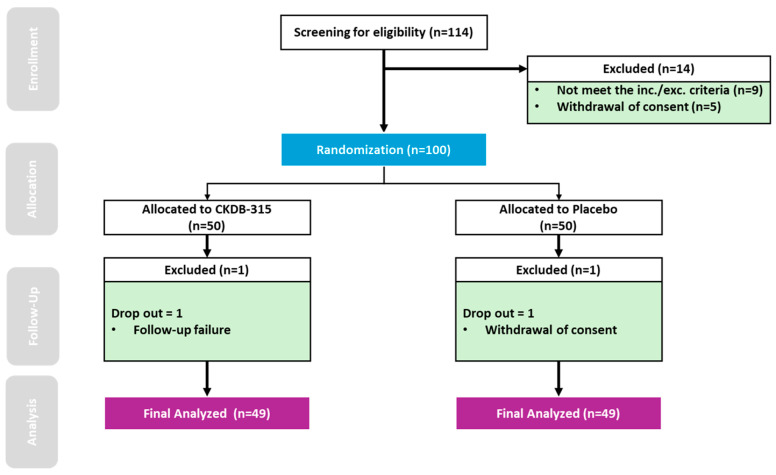
Flowchart of Screening, Randomization, and Study Completion.

A flowchart of the study protocol is shown in Figure 1. Of the 114 screened participants, 100 were randomized into the CKDB-315 (n = 50) and placebo (n = 50) treatment groups. All randomized participants were included in the safety analysis. Of these, 98 were included in the FAS after excluding two participants owing to efficacy assessment omissions (CKDB-315 group: n = 49, placebo group: n = 49). The baseline demographic characteristics were balanced, with no significant differences observed between groups (Table 1).

### 3.2. Efficacy Outcomes

#### 3.2.1. Changes in Health-Related Quality of Life; SGRQ and CAT

**Figure 2:**
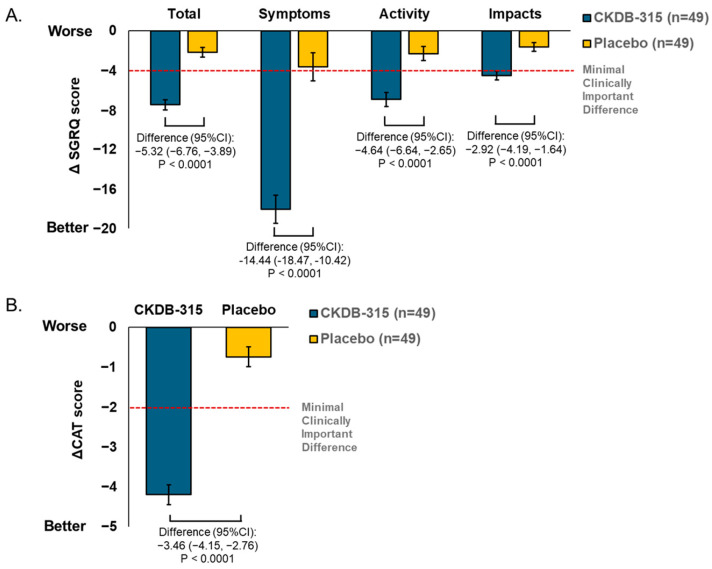
Comparison of the mean changes from baseline to 12 weeks in (**A**) SGRQ scores (Total, Symptoms, Activity, and Impacts) and (**B**) CAT total score between the CKDB-315 group and the placebo group using the least squares (LS) method. The *p*-values are calculated from ANCOVA tests that have been corrected for baseline values. SGRQ is for St. George’s Respiratory Questionnaire, CAT stands for Chronic Obstructive Pulmonary Disease Assessment Test, and CI stands for confidence interval.

**Figure 3:**
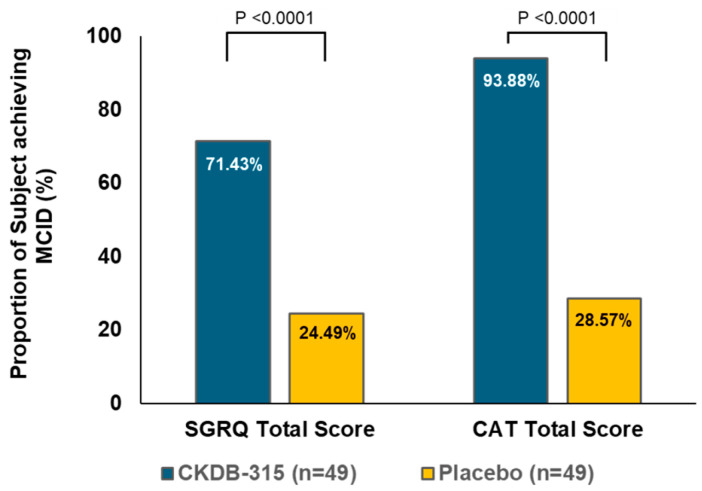
A comparison was made between the CKDB-315 group and the placebo group to determine the proportion of participants who reached the minimum clinically important difference (MCID) for changes in the SGRQ total score and CAT score after 12 weeks. The minimum clinically important difference (MCID) was defined as a decrease of 4 points from the initial value for the St. George’s Respiratory Questionnaire (SGRQ) total score and a decrease of 2 points from the initial value for the COPD Assessment Test (CAT) total score. The comparison between the groups was analyzed using Pearson’s chi-square test. MCID is an acronym for Minimal Clinically Important Difference. SGRQ stands for St. George’s Respiratory Questionnaire, while CAT is an abbreviation for Chronic Obstructive Pulmonary Disease Assessment Test.

At 12 weeks, CKDB-315 supplementation significantly improved the least squares (LS) mean change in St. George’s Respiratory Questionnaire (SGRQ) total scores compared with the placebo (95% CI for LS mean difference between groups, −5.32; 95% CI: −6.76–−3.89; *p* < 0.0001) (Figure 2A). Improvements in LS mean changes were consistently greater with CKDB-315 supplementation than with the placebo in all SGRQ domains (Symptoms, Activity, and Impacts) from baseline to 12 weeks (Figure 2A). Similarly, CKDB-315 supplementation significantly improved the LS mean change in the COPD Assessment Test (CAT) score compared with the placebo (95% CI for LS mean difference between groups, −3.46; 95% CI: −4.15–−2.76; *p* < 0.0001) (Figure 2B). The number of participants who achieved a minimum clinically important difference (MCID) (a 4-point decrease from baseline) for change in the SGRQ total score at 12 weeks was significantly greater in the CKDB-315 group than in the placebo group (71.43% vs. 24.49%; *p* < 0.0001) (Figure 3). Additionally, positive outcomes were observed in the participants who achieved the MCID (a 2-point decrease from baseline) for the change in the CAT total score at 12 weeks (93.88% in the CKDB-315 group vs. 28.57% in the placebo group; *p* < 0.0001) (Figure 3).

#### 3.2.2. Changes in Dyspnea

**Figure 4:**
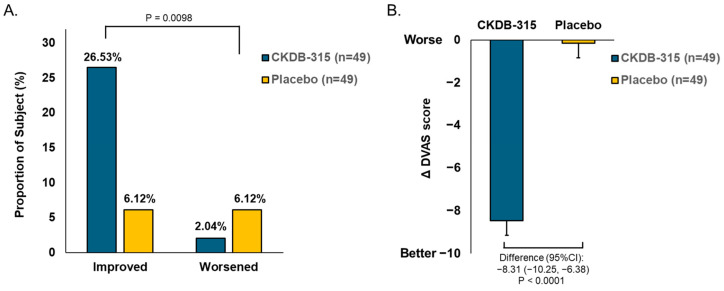
Comparison of dyspnea changes from baseline to 12 weeks between the CKDB-315 group and the placebo group. (**A**) The changes on the mMRC dyspnea scale were categorized as follows: participants who exhibited a decrease of one grade were defined as ‘Improved’, whereas those who had an increase of one grade were labeled as ‘worsened’. (**B**) The LS mean changes in the DVAS are being referred to. The *p*-values for the differences in categorical changes in mMRC across groups were computed using Fisher’s exact test. The *p*-values for the differences in least squares mean changes in the DVAS across the groups were obtained using ANCOVA tests that were corrected for the baseline values. Abbreviations: mMRC is for modified Medical Research Council, DVAS stands for dyspnea visual analogue scale, LS stands for least squares, and CI stands for confidence interval.

A significant difference between groups was observed in the categorized change in mMRC scores from baseline to week 12 (*p* = 0.0098). The number of participants with improved mMRC scores was higher in the CKDB-315 group than in the placebo group (26.53% vs. 6.12%). However, that of participants with worsening scores was lower in the CKDB-315 group than in the placebo group (2.04% vs. 6.12%) (Figure 4A). Similarly, the mean LS change in the dyspnea visual analog scale (DVAS) significantly improved from baseline to 12 weeks in the CKDB-315 group compared with that in the placebo group (*p* < 0.0001) (Figure 4B).

#### 3.2.3. Changes in Pulmonary Function Values

Analysis of the mean LS changes in pulmonary function values (forced vital capacity, FVC; predicted FVC%; forced expiratory volume in one second, FEV1; predicted FEV1%; and FEV1/FVC) from baseline to 12 weeks revealed no statistically significant differences between the groups (*p* > 0.05) (Table S1).

#### 3.2.4. Changes in TAS and Inflammatory Cytokine Levels

After 12 weeks of treatment, the total antioxidant status (TAS) level significantly increased with CKDB-315 supplementation compared to the placebo (*p* = 0.0351) (Figure S1). Moreover, inflammatory cytokine levels improved. CKDB-315 supplementation significantly reduced the mean LS change in hs-CRP compared to the placebo (95% CI for mean LS difference between groups, −1.35; 95% CI: −2.62–−0.08; *p* = 0.0372) (Figure S2A). The mean change in IL-6 levels was significantly reduced from baseline in the CKDB-315 group (−0.25 pg/mL; *p* = 0.0262 within the group, with a marginally significant difference between groups (*p* = 0.0520) (Figure S2B). Furthermore, the mean change in TNF-α values considerably decreased in the CKDB-315 group (−0.04 pg/mL; *p* = 0.0547 within the group. However, no significant difference was observed between groups (*p* = 0.2375) (Figure S2C).

### 3.3. Safety

The incidence of adverse events was 4.00% (n = 2) and 8.00% (n = 4) in the CKDB-315 and placebo groups, respectively. However, no significant differences were observed between the groups (*p* > 0.05). Furthermore, no drug-related, serious, or serious drug-related adverse events were observed (Table S2). Additionally, no clinically significant changes were observed in the physical examinations, vital signs, or clinical laboratory test results of participants in the two intervention groups.

### 3.4. Exploratory Outcomesof Microbiome Analyses

After 12 weeks of treatment, we performed an analysis targeting the Full Analysis Set (FAS) to examine the gut microbiota and metabolites, particularly short-chain fatty acids. Ninety-three participants were included in the gut microbiota analysis (48 and 45 in the CKDB-315 and placebo groups, respectively). However, seven participants were excluded: two due to ‘dropout’ (n = 1 per group), one from the placebo group due to ‘sample not collected’, and four due to ‘insufficient sample quantity’ (one and three from the CKDB-315 and placebo groups, respectively). Ninety-seven participants were initially included for the short-chain fatty acid analysis (49 and 48 in the CKDB-315 and placebo groups, respectively). Two participants (n = 1 per group) were excluded due to ‘dropout’, and one participant from the placebo group was excluded due to ‘sample not collected’.

**Figure 5:**
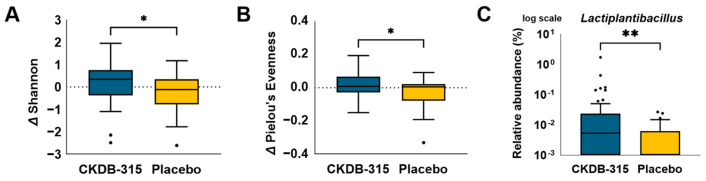
Comparison of the alterations in the intestinal microbiota between the CKDB-315 group (n = 48) and the placebo group (n = 45) from baseline to 12 weeks. (**A**) Assessment of Shannon’s diversity index; (**B**) evaluation of Pielou’s evenness index; (**C**) determination of the relative abundance of the *Lactiplantibacillus* genus. The comparison between groups was examined using the Wilcoxon rank sum test. A single asterisk (*) indicates significant difference at *p* < 0.05, and double asterisk (**) indicated significant difference at *p* < 0.001.

Our analysis of the gut microbiota demonstrated a significant increase in α-diversity in the CKDB-315 group compared to the placebo group (Shannon’s diversity index, *p* = 0.0305; Pielou’s evenness index, *p* = 0.0336) (Figure 5A,B). Moreover, the overall microbial composition analysis revealed a considerable increase in the distribution of the *Lactiplantibacillus* genus (Figure 5C). The concentrations of propionic and butyric acids, which maintain gut homeostasis and activate various immune cells, were markedly elevated in the CKDB-315 group (Table 2).

## 4. Discussion

This study aimed to investigate the efficacy and safety of CKDB-315 in adults with respiratory discomfort in a rigorous multicenter, randomized, double-blind, placebo-controlled clinical trial. Our findings provide valuable insights into the potential therapeutic benefits of CKDB-315 against respiratory symptoms and in overall health outcomes.

Particulate matter (PM) refers to solid and liquid particles that are released directly into the air from various sources, including diesel, road and agricultural dust, and industrial activities. These particles remain suspended in the air, causing air pollution [19]. PM has different impacts on the environment and human health, depending on its morphological, chemical, physical, and thermodynamic properties [20]. Particles <10 μm can penetrate the respiratory tract and affect gas exchange in the lungs, whereas those <1 μm can act like gas molecules and penetrate the alveoli and circulatory system [21]. The most toxic components of PM2.5 include metals, polycyclic aromatic hydrocarbons, carbonaceous particles, and other organic compounds [22]. Transition metals in PM, particularly iron, can increase the production of reactive oxygen species, causing inflammation and tissue damage [23]. Levels of cytokines such as TNF-α, IP-10, MCP-1, IL-6, and IL-1β are elevated in response to PM2.5 exposure, indicating a pro-inflammatory state [24]. Thus, PM can worsen respiratory symptoms and cause decreased lung function, especially in patients with lung diseases such as COPD and asthma [25]. Long-term exposure to outdoor PM decreased FEV1 in patients with severe COPD. Higher PM concentrations within 24 h increased the same-day and 1-day delayed peak expiratory flow, resulting in decreased FEV1 [26].

Primary efficacy analysis based on the FAS revealed significant improvements in respiratory health parameters among participants receiving CKDB-315 compared to those receiving the placebo. CKDB-315 reduced the total SGRQ and CAT scores more effectively than the placebo, highlighting its potential as a treatment option for respiratory disorders. The percentage of participants who reached an MCID in the SGRQ total score was 71.43% and 24.49% in the CKDB-315 and placebo groups, respectively. In contrast, that in the CAT total score was 93.88% and 28.57% in the CKDB-315 and placebo groups, respectively. These findings underscore the clinical utility of CKDB-315 in the improvement of respiratory symptoms, activity levels, and the overall quality of life in patients with respiratory disorders. Moreover, secondary efficacy outcomes further demonstrated the favorable effects of CKDB-315; individual SGRQ and CAT scores, as well as the DVAS were significantly reduced. Pulmonary function tests provided additional insights into the effects of CKDB-315 on respiratory function, enhancing our understanding of its therapeutic potential against respiratory discomfort. Both groups showed a decrease in the FEV1/FVC ratio, suggesting potential limitations in airway function, despite no significant differences in FEV1 or predicted FEV1%. The recruitment of individuals with normal lung function in this trial resulted in the limited assessment of changes in lung volume within and between the groups. Nevertheless, conducting the trial in individuals with respiratory discomfort despite normal lung function revealed significant improvements. Thus, CKDB-315 can be considered a valuable substance, potentially contributing to improvements even in individuals with abnormal lung function.

Exploratory analyses focusing on the gut microbiota and short-chain fatty acids elucidated the potential mechanisms underlying the therapeutic effects of CKDB-315. The composition of the gut microbiota was significantly altered, with an increase in α-diversity and abundance of the *Lactiplantibacillus* genus in the CKDB-315 group. These changes may contribute to the modulation of immune responses and inflammation, thereby affecting respiratory health outcomes. Additionally, elevated levels of short-chain fatty acids such as propionic acid and butyric acid in the CKDB-315 group suggested potential benefits for gut homeostasis and immune regulation [27], which could indirectly influence respiratory function [27]. Furthermore, comparison of changes in the levels of inflammatory cytokines between the treatment groups from baseline to 12 weeks revealed significantly decreased hs-CRP in the CKDB-315 group compared to that in the placebo group (Figure S2). Inflammatory cytokines, including TNF-α, and IL-6, have significant involvement in the pathogenesis of respiratory illnesses. TNF-α is a cytokine with pro-inflammatory properties that has a role in the inflammatory response and tissue damage associated with respiratory diseases [28]. IL-6, a crucial cytokine, has a role in the immediate response to infection and is linked to the seriousness of respiratory infections and chronic illnesses [29]. IL-1β plays a critical role in the inflammatory response of respiratory diseases by promoting the release of other pro-inflammatory cytokines and mediators, thereby contributing to airway inflammation and tissue damage. IL-8 is a key chemokine involved in the recruitment and activation of neutrophils, leading to increased inflammation and exacerbation of conditions such as chronic obstructive pulmonary disease (COPD) and asthma [28,29]. The decrease in these cytokines seen with the addition of CKDB-315 indicates its potential effectiveness in regulating inflammation and enhancing respiratory well-being. The notable decline in hs-CRP and IL-6 levels, together with the inclination towards decreased TNF-α levels, suggests that CKDB-315 may have potential benefits in the management of respiratory illnesses associated with inflammation.

Our safety analysis demonstrated that CKDB-315 was well tolerated, with a low incidence of adverse events compared to the placebo. No serious or drug-related adverse events were reported. Similarly, no clinically significant changes were observed in physical examination or laboratory parameters, indicating a favorable safety profile for CKDB-315. Both asthma and COPD are chronic respiratory diseases characterized by inflammatory changes in the respiratory tract [30]. Asthma is the 16th leading cause of years lived with disability. Moreover, the prevalence of asthma in adolescents aged 13–14 years is between 6 and 27%, which has a significant impact on the young population [31]. COPD is associated with a high disease burden and is predicted to become the third leading cause of death by 2030 [32]. Steroids are used to treat chronic respiratory diseases. However, various side effects may occur in patients who are administered high or standard doses of steroids for long periods. Inhaled corticosteroid (ICS) therapy used to treat asthma can cause adrenal insufficiency by suppressing the hypothalamic–pituitary–adrenal axis [33,34]. This therapy is associated with skin thinning, increased bruising, and hirsutism in children. Even low to moderate doses may reduce growth rate in children with asthma when administered regularly [35]. ICS may increase the risk of pneumonia in patients with COPD [36,37]. Additionally, the long-term use of this therapy in patients with COPD increases the risk of osteoporosis and fractures [38]. Therefore, it is crucial to develop functional foods that prevent CRD and alleviate respiratory symptoms to eliminate the risks associated with long-tern ICS usage. This study demonstrated that CKDB-315 is a safe functional food option for CRD prevention, confirming its potential use.

CKDB-315 is a compound comprising the probiotic strain *L. plantarum* KC3 and the LJH extract. *L. plantarum* KC3 is a native bacterial strain found in fermented kimchi, whereas LJH is a natural extract obtained from Korean motherwort, which possesses anti-inflammatory and antioxidant properties [15,18]. A previous study revealed the superior activity of *L. plantarum* KC3 combined with LJH extract compared with treatment with individual components, suggesting its potential as a functional material for improving respiratory health [18].

Our findings suggest that CKDB-315 provides promising therapeutic benefits for respiratory health. It may exert beneficial effects on respiratory health by modulating immune response, reducing inflammation, and strengthening respiratory mucosal barrier function. *L. plantarum* KC3 alleviates respiratory symptoms and improves overall lung function by promoting a balanced microbial environment and strengthening host defenses [15,18]. LJH extract, which is traditionally used for its anti-inflammatory and bronchodilator properties, has antioxidant and anti-inflammatory effects that may help alleviate airway inflammation and oxidative stress associated with respiratory diseases [18]. This dilating effect may contribute to improved airway function and symptom relief in individuals with respiratory diseases.

## 5. Conclusions

In conclusion, CKDB-315, a novel combination of *Lactiplantibacillus plantarum* KC3 and *Leonurus japonicus* Houtt. extracts, demonstrated significant improvements in respiratory symptoms, quality of life, and overall health outcomes in adults with respiratory discomfort. This multicenter, randomized, double-blind, placebo-controlled clinical trial provided robust evidence supporting the efficacy and safety of CKDB-315. The primary and secondary efficacy outcomes indicated that CKDB-315 effectively reduced SGRQ and CAT scores, improved dyspnea, and enhanced antioxidant status, suggesting its potential as a therapeutic option for respiratory health. Exploratory analyses further revealed beneficial changes in gut microbiota composition and short-chain fatty acid levels, which may contribute to the observed health benefits. Importantly, CKDB-315 was well tolerated with no significant adverse events reported, underscoring its safety profile.

Given the increasing prevalence of chronic respiratory diseases and the limitations of current therapeutic options, CKDB-315 offers a promising alternative for managing respiratory discomfort. Further research is warranted to confirm these findings in larger and more diverse populations and to elucidate the underlying mechanisms of action. These findings suggest that CKDB-315 could be a valuable addition to the therapeutic arsenal for respiratory health, potentially improving the quality of life for individuals suffering from chronic respiratory conditions.

## Figures and Tables

**Table 1:** Baseline characteristics of subjects (Full Analysis Set).

	CKDB-315 (n = 49)	Placebo (n = 49)	*p*-Value
Sex			0.6018
Male, n (%)	8 (16.33)	10 (20.41)	
Female, n (%)	41 (83.67)	39 (79.59)	
Age, yr	50.49 ± 11.45	50.14 ± 8.37	0.8757
BMI, kg/m^2^	24.37 ± 2.93	24.42 ± 2.42	0.9253
History of respiratory symptoms			
Past, n (%)	0 (0.00)	0 (0.00)	-
Present, n (%)	49 (100.00)	49 (100.00)	-
Cough, n (%)	47 (95.92)	43 (87.76)	0.2682
Duration, months	49.37 ± 63.94	47.80 ± 72.77	0.0810
Sputum, n (%)	47 (95.92)	49 (100.00)	0.4948
Duration, months	57.19 ± 66.94	66.65 ± 101.31	0.2469
Dyspnea, n (%)	22 (44.90)	21 (42.86)	0.8387
Duration, months	45.13 ± 32.40	64.69 ± 111.70	0.8269
History of smoking			0.6171
Non, n (%)	46 (93.88)	48 (97.96)	
Past, n (%)	3 (6.12)	1 (2.04)	
Present, n (%)	0 (0.00)	0 (0.00)	
History of drinking			0.4527
Non, n (%)	40 (81.63)	37 (75.51)	
Past, n (%)	1 (2.04)	0 (0.00)	
Present, n (%)	8 (16.33)	12 (24.49)	

Data are presented as the mean ± standard deviation or number (%), unless otherwise specified. *p*-values were calculated using the independent *t*-test or Wilcoxon rank-sum test for continuous variables depending on the normality of data in each group and chi-squared test or Fisher’s exact test for categorical variables. BMI, Body mass index.

**Table 2:** Change in short-chain fatty acids in fecal samples from baseline to 12 weeks.

	CKDB-315 (n = 49)	*p*-Value	Placebo (n = 48)	*p*-Value
Acetic acid (μmol/g)	−0.54 ± 36.01	0.5569	−2.28 ± 28.62	0.5829
Butyric acid (μmol/g)	4.79 ± 25.55	0.0468	4.80 ± 19.98	0.1026
Propionic acid (μmol/g)	12.13 ± 36.81	0.0048	6.59 ± 28.43	0.3216

Data are presented as the mean ± standard deviation, unless otherwise specified. *p*-values were calculated using the Wilcoxon’s signed rank test or Paired *t*-test depending on the normality of data in each group for within-group comparison.

## Data Availability

Data are contained within the article and Supplementary Materials.

## Supplementary Materials

The following supporting information can be downloaded at: https://www.mdpi.com/article/10.3390/nu16132128/s1. Figure S1: Comparison of least squares mean increases in total antioxidant status (TAS) from baseline to 12 weeks between the CKDB-315 group and the placebo group; Figure S2: Comparison of the least squares mean change from baseline to 12 weeks in high-sensitivity C-reactive protein (hs-CRP), interleukin-6 (IL-6), and tumor necrosis factor-alpha (TNF-α) between the CKDB-315 group and the placebo group; Table S1: LS mean changes in pulmonary function values from baseline to 12 weeks; Table S2: Adverse events.

## Abbreviations

AE	Adverse Event
ALT	Alanine Transaminase
AST	Aspartate Transaminase
BMI	Body Mass Index
CAT	COPD Assessment Test
COPD	Chronic Obstructive Pulmonary Disease
CRDs	Chronic Respiratory Disease
DVAS	Dyspnea Visual Analogue Scale
FAS	Full Analysis Set
FEV1	Forced Expiratory Volume in 1 s
FVC	Forced Vital Capacity
GCP	Good Clinical Practice
HRQL	Health-Related Quality of Life
hs-CRP	High-Sensitivity C-Reactive Protein
ICS	Inhaled Corticosteroid
IL-6	Interleukin-6
ITT	Intention-to-Treat Principle
LJH	*Leonurus Japonicus* Houtt.
LS	Least Squares
MCID	Minimal Clinically Important Difference
MedDRA	Medical Dictionary for Regulatory Avtivities
mMRC	modified Medical Research Council Dyspnea Scale
PT	Preferred Term
SCFAs	Short-Chain Fatty Acids
SGRQ	St. George’s Respiratory Questionnaire
SOC	System Organ Class
SS	Safety Analysis Set
TAS	Total Antioxidant Status
TNF-α	Tumor Necrosis Factor-α
ULN	Upper Limit of Normal
