# Supplementary material for: Effect of a Combination of Lactiplantibacillus plantarum KC3 and Leonurus japonicus Extracts in Respiratory Discomfort: A Randomized, Double-Blind, Placebo-Controlled Trial
Source: Nutrients. 2024 Jul 3;16(13):2128. doi: 10.3390/nu16132128 (PMC11243513; doi:10.3390/nu16132128)
Supplement: Supplementary file 1 [file nutrients-16-02128-s001.zip › nutrients-3067653-supplementary.pdf]

nutrients

MDPI

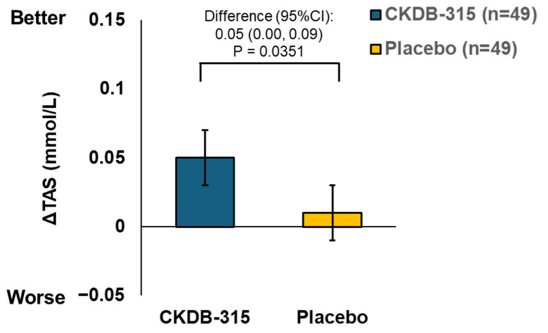

Figure S1. Comparison of least squares mean increases in total antioxidant status (TAS) from baseline to 12 weeks between the CKDB-315 group and the placebo group. The *p*-values were computed using ANCOVA tests that were adjusted for baseline values. Abbreviations: TAS is for Total Antioxidant Status, LS stands for Least Squares, and CI stands for Confidence Interval.

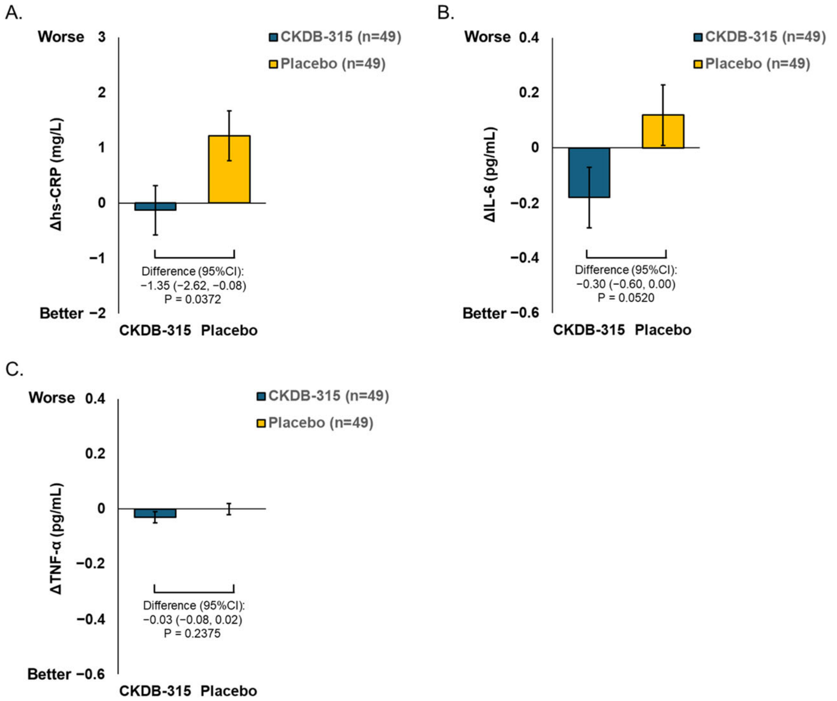

Figure S2. Comparison of the least squares mean change from baseline to 12 weeks in high-sensitivity C-reactive protein (hs-CRP), interleukin-6 (IL-6), and tumor necrosis factor-alpha (TNF-α) between the CKDB-315 group and the placebo group. The *p*-values were calculated from ANCOVA tests that have been corrected for baseline values. hs-CRP refers to high sensitivity C-reactive protein, IL-6 stands for interleukin 6, TNF-α represents tumor necrosis factor-α, LS is an abbreviation for Least Squares, and CI is for Confidential Interval.

Table S1. LS mean changes in pulmonary function values from baseline to 12 weeks.

	CKDB-315 (n=49)	Placebo (n=49)	*p*-Value
FVC (L)	0.04±0.03	0.06±0.03	0.4625
predicted FVC (%)	1.56±0.74	1.44±0.74	0.9128
FEV1 (L)	-0.01±0.02	0.00±0.02	0.7196
predicted FEV1 (%)	0.24±0.68	-0.30±0.68	0.5719
FEV1/FVC (%)	-1.27±0.41	-1.58±0.41	0.5961

Data are presented as the LS mean ± standard error. *p*-values were derived from ANCOVA tests adjusted for baseline values. FVC, forced vital capacity; FEV1, forced expiratory volume in one second; LS, Least Squares; CI, Confidential Interval

Table S2. Adverse events.

	CKDB-315 (n = 50)	Placebo (n = 50)
Treatment Emergent Adverse event	2 (4)	4 (8)
Preferred term		
COVID-19	2 (4)	1 (2)
Nasopharyngitis	0	1 (2)
Otolithiasis	0	1 (2)
Chills	0	1 (2)
Blood cholesterol increased	0	1 (2)
Low density lipoprotein increased	0	1 (2)
Adverse Drug Reaction	0	0
Serious Adverse Event	0	0
Serious Adverse Drug Reaction	0	0

Data are presented as the number (%). Adverse events were coded according to the preferred term of the MedDRA Version 26.0. MedDRA, Medical Dictionary for Regulatory Activities
